# Posterior thoracolumbar hemivertebra resection and short-segment fusion in congenital scoliosis: surgical outcomes and complications with more than 5-year follow-up

**DOI:** 10.1186/s12893-021-01165-8

**Published:** 2021-03-25

**Authors:** Beixi Bao, Qingjun Su, Yong Hai, Peng Yin, Yaoshen Zhang, Shiqi Zhu, Zhencheng Sun

**Affiliations:** 1 Department of Orthopedic Surgery, Beijing Tongren Hospital, Capital Medical University, No. 1 of Dongjiaominxiang Street, Dongcheng District, Beijing, 100730 China; 2 Department of Orthopedic Surgery, Beijing Chaoyang Hospital, Capital Medical University, 8 Gong Ti Nan Lu, Chaoyang District, Beijing, 100020 China

**Keywords:** Congenital scoliosis, Thoracolumbar hemivertebra, Hemivertebra resection, Pedicle screw fixation

## Abstract

**Background:**

Treatment of congenital hemivertebra is challenging and data on long-term follow-up (≥ 5 years) are lacking. This study evaluated the surgical outcomes of posterior thoracolumbar hemivertebra resection and short-segment fusion with pedicle screw fixation for treatment of congenital scoliosis with over 5-year follow-up.

**Methods:**

This study evaluated 27 consecutive patients with congenital scoliosis who underwent posterior thoracolumbar hemivertebra resection and short-segment fusion from January 2007 to January 2015. Segmental scoliosis, total main scoliosis, compensatory cranial curve, compensatory caudal curve, trunk shift, shoulder balance, segmental kyphosis, and sagittal balance were measured on radiographs. Radiographic outcomes and all intraoperative and postoperative complications were recorded.

**Results:**

The segmental main curve was 40.35° preoperatively, 11.94° postoperatively, and 13.24° at final follow-up, with an average correction of 65.9%. The total main curve was 43.39° preoperatively, 14.13° postoperatively, and 16.06° at final follow-up, with an average correction of 60.2%. The caudal and cranial compensatory curves were corrected from 15.78° and 13.21° to 3.57° and 6.83° postoperatively and 4.38° and 7.65° at final follow-up, with an average correction of 69.2% and 30.3%, respectively. The segmental kyphosis was corrected from 34.30° to 15.88° postoperatively and 15.12° at final follow-up, with an average correction of 61.9%. A significant correction (p < 0.001) in segmental scoliosis, total main curve, caudal compensatory curves and segmental kyphosis was observed from preoperative to the final follow-up. The correction in the compensatory cranial curve was significant between preoperative and postoperative and 2-year follow-up (p < 0.001), but a statistically significant difference was not observed between the preoperative and final follow-up (p > 0.001). There were two implant migrations, two postoperative curve progressions, five cases of proximal junctional kyphosis, and four cases of adding-on phenomena.

**Conclusion:**

Posterior thoracolumbar hemivertebra resection after short-segment fusion with pedicle screw fixation in congenital scoliosis is a safe and effective method for treatment and can achieve rigid fixation and deformity correction.

## Background

Congenital scoliosis is caused by spine abnormalities that lead to imbalanced growth. A frequent cause is a single hemivertebra as a unilateral failure of formation. Because of local deformity and asymmetric loads, unaffected neighboring vertebrae subsequently show asymmetric growth. This congenital anomaly, apart from causing adverse effects on cardiopulmonary function, also causes psychological distress [1]. Nonsurgical treatment for hemivertebra is seldom successful; 75% of the curves are progressive and only 5–10% can be treated with bracing [2]. Early surgical intervention can mitigate deformity progression and psychological stress [3]. Surgical treatment modalities include in situ posterior spinal fusion, combined anterior and posterior in situ spinal fusion, hemivertebra resection [4], and guided growth procedures such as growing rods [5], Vertical Expandable Prosthetic Titanium Rib (VEPTR, SYNTHES Spine) [6], and Shilla [7] procedures.

Posterior thoracolumbar hemivertebra resection and short-segment fusion provides satisfactory deformity correction and rigid stabilization, with markedly less surgical time and fewer complications than other procedures [8]. The first long-term follow-up of postsurgical outcomes for this treatment was reported by Chang et al. in 2015 [9], for 18 congenital scoliosis patients with a mean follow-up of 11.4 years; however, the hemivertebrae location was not reported. Overall, long-term follow-up data are limited for this surgery. Thoracolumbar region was the transition area between fixed thoracic segment and relatively motional lumbar segment, where facet joints change from coronal to sagittal plane and the spine alignment change from lordotic to kyphotic form. Therefore, this study aimed to determine surgical outcomes of posterior thoracolumbar hemivertebrae resection and short-segment fusion with pedicle screw fixation for treatment of congenital scoliosis with a minimum of 5-year follow-up. Radiographic outcomes as well as intraoperative and postoperative complications were recorded. We believe that our study of the clinical efficacy of thoracolumbar hemivertebra resection and short segment fixation is of guiding significance for clinical treatment.

## Methods

This retrospective study analyzed 27 consecutive patients with congenital scoliosis secondary to thoracolumbar hemivertebra who underwent posterior thoracolumbar single hemivertebra resection; transpedicular, short-segment fixation; and fusion from January 2007 to January 2015 with over 5-year follow-up. The corresponding author performed all surgeries. All patients were advised regarding possible surgical outcomes and signed written informed consent before surgery. The institutional review board of the authors’ hospital approved this study.

Inclusion criteria were (1) congenital spinal deformity caused by a single hemivertebra requiring surgical treatment (curve magnitude: > 25° and/or with fast progression, defined as documented progression of the curve of > 5° in 6-month follow-up and/or failure of conservative treatment); (2) hemivertebra located in thoracolumbar (T–L) region (T11–L2); (3) short-segment fixation and fusion (≤ 6 levels); and (4) > 5 years of radiographic follow-up after initial surgery. Exclusion criteria were (1) anterior approach, (2) operative approach using hooks as anchors, (3) > 1 hemivertebra or in combination with other congenital spinal deformities, (4) congenital kyphosis without scoliosis, and (5) previous spinal surgery.

**Fig. 1:**
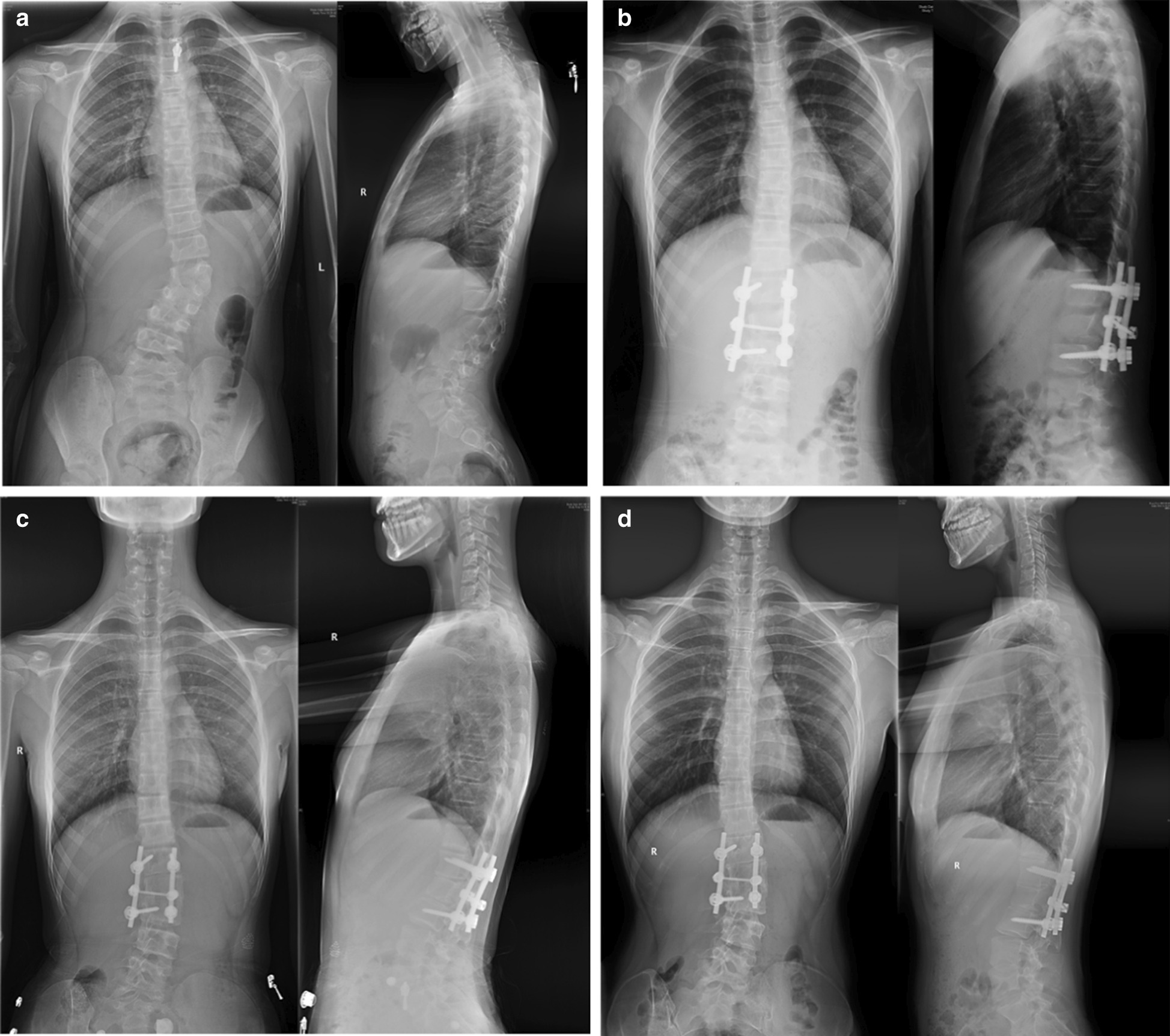
Radiograph of a patient with congenital scoliosis. A hemivertebra is located at L2 (second lumbar vertebra). The standard standing long cassette anterior–posterior and lateral radiographs were performed preoperatively (**a**), postoperatively (**b**), at 2-year follow-up (**c**), and at the 10-year follow-up visit (July 8, 2019) (**d**). The patient demonstrated the adding-on phenomenon at 2-year follow-up

Standard standing long-cassette anterior–posterior and lateral radiographs were performed preoperatively, postoperatively, at 2-year follow-up, and at final follow-up (minimum of 5 years postoperatively) (Fig. 1). Operative reports and medical records were reviewed for operative and demographic data and any complications.

### Radiographic assessment

**Fig. 2:**
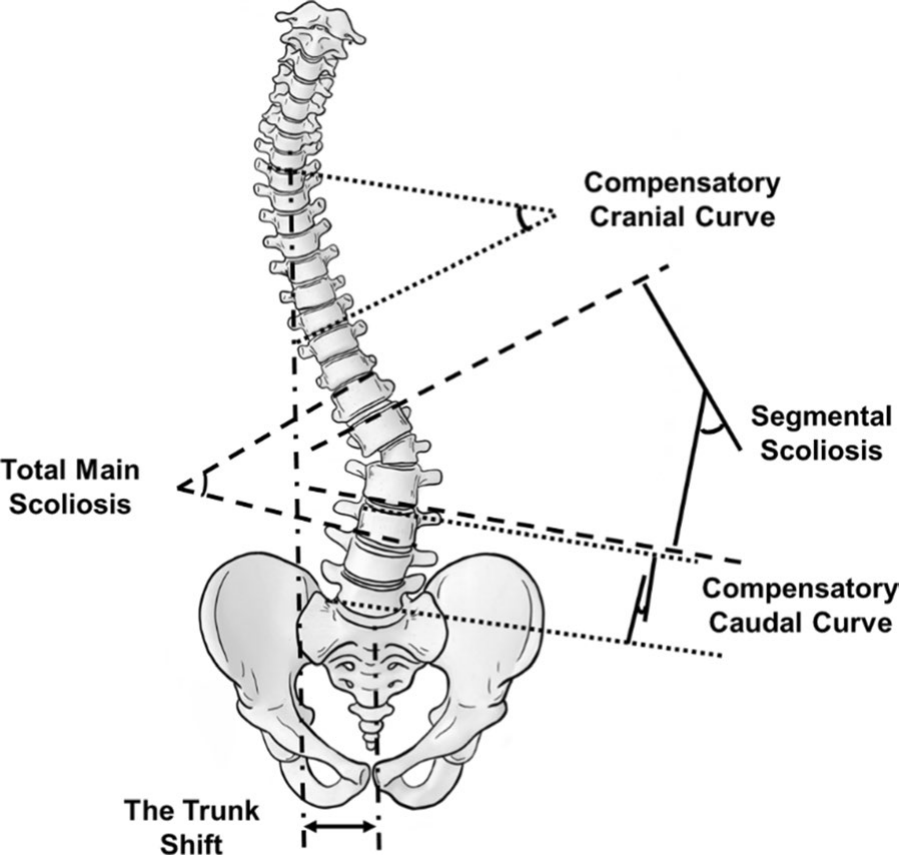
Angles in the coronal plane: segmental scoliosis, total main scoliosis, compensatory cranial curve, compensatory caudal curve, and trunk shift

**Fig. 3:**
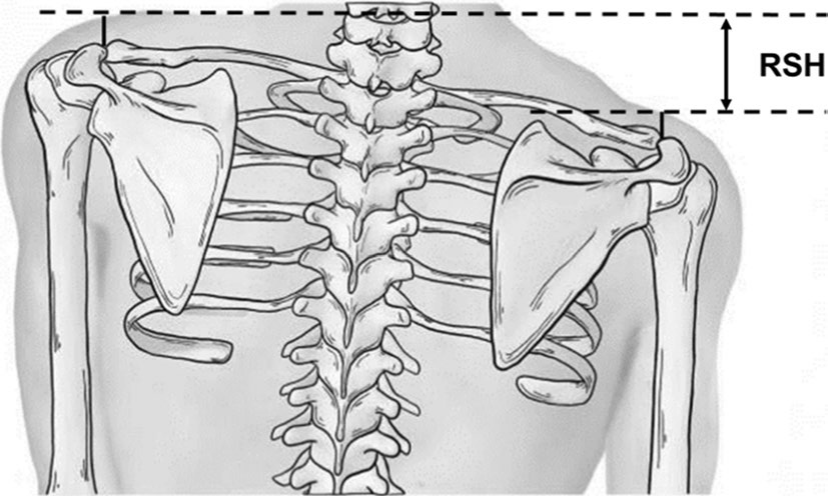
Measurement in the coronal plane: shoulder balance, defined by radiographic shoulder height (RSH)

Whole-spine radiographs were reviewed by an independent observer to measure deformity. Curves measured in the coronal plane (Figs. 2 and 3) included segmental scoliosis, total main scoliosis, compensatory cranial curve, compensatory caudal curve, trunk shift, and shoulder balance. Segmental scoliosis was measured from the upper vertebral endplate above the hemivertebra to the lower endplate below the hemivertebra. Total main scoliosis was the maximum scoliosis angle between the two most tilted vertebrae. Compensatory cranial curve and the compensatory caudal curve were also measured. Trunk shift was measured as the perpendicular distance from the sacrum center to the plumb line drawn from the midpoint of the seventh cervical vertebra (C7). Shoulder balance was evaluated using radiographic shoulder height, defined as the difference in soft tissue shadows directly superior to acromioclavicular joints on both sides, designated positive (+) when the left shoulder was higher and negative (−) when the right shoulder was higher.

**Fig. 4:**
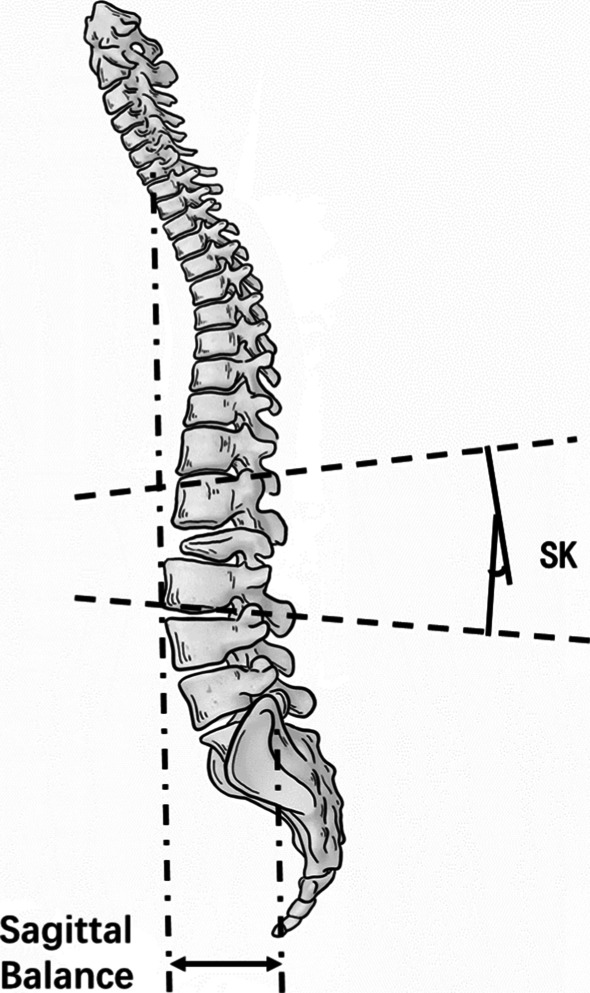
Measurement in the sagittal plane: segmental kyphosis (SK) and sagittal balance

Segmental kyphosis (SK) and sagittal balance were measured in the sagittal plane (Fig. 4). SK was measured from the upper endplate above the hemivertebra to the lower endplate below the hemivertebra. Sagittal balance was measured as the distance from the C7 plumb line to a perpendicular line drawn from the posterosuperior corner of the sacrum, designated as positive (+) when the plumb line was anterior and negative (−) when posterior to the posterosuperior corner of the sacrum [10].

### Surgical technique

Hemivertebra resection was by the single-stage posterior approach with fusion of the immediately adjacent vertebrae using transpedicular instrumentation [10]. A standard midline incision was created with patients in the prone position. Pedicle screws were inserted into normal upper/lower vertebrae. A pre-contoured rod was connected to the screws on the convex side. Posterior hemivertebra elements and adjacent levels were removed to expose the pedicle and nerve roots above and below. Resection of posterior elements was performed at the end to protect the dural sac and cord. The anterior hemivertebra was exposed using blunt dissection. A wedge osteotomy was performed from the convex to concave side. Using a rongeur, osteotome, curette, and abrasive drilling, the anterior vertebral body was hollowed. Upper and lower discs, including cartilage endplate, were removed from the bleeding bone. Gradual compression was applied while leaving the concave rod unlocked until the gap was closed. Anterior reconstruction with a titanium mesh cage was performed if large hemivertebra complicated the gap closure by compression. If high compressive force was necessary to correct deformity, especially pronounced kyphosis, one to two additional segments were permanently or temporarily included in instrumentation to avoid overloading the pedicles and thereby causing pedicle fracture. Caution was taken to ensure that exiting nerve roots and dura were not impinged. Finally, decortication of posterior elements was performed. Autologous bone from the hemivertebra was used for posterolateral fusion.

Curve progression was defined a curve newly developed with a > 20° increase in the Cobb angle, with the apical vertebra located ≥ 2 levels from the lower instrumented vertebra. The most common diagnostic criteria of adding-on were (1) progressive increase in the number of vertebrae included within the distal curve, (2) with either > 5 mm increase in the first vertebra deviation below the instrumentation from the center sacral vertical line (CSVL), or (3) > 5° increase of angulation of the first disc below the instrumentation [11]. Another study considered that diagnostic criteria for adding-on phenomenon should include the Cobb angle increase in the lumbar curve and a minimum 1 year follow-up [12]. Proximal junctional kyphosis (PJK) is defined as a proximal junctional sagittal Cobb angle of ≥ 10° or a proximal junctional sagittal Cobb angle ≥ 10° than the preoperative measurement [13].

### Statistical analysis

One author was an independent observer who completed the evaluation procedure in a blinded manner. Statistical data were analyzed using the Statistical Package for Social Sciences (SPSS, version 20, IBM) software. Continuous data were expressed as mean and standard deviation. Correction rates were expressed as percentages. Hypothesis testing relied on repeated measurement design data variance analysis.

## Results

The study included 27 patients: 11 male and 16 female. The mean age at the time of surgery was 11 years (5–16 years). The average follow-up period was 68 months (60–122 months). All patients reached skeletal maturity at the final follow-up. The mean operative time was 161 min (60–300 min), and the mean volume of intraoperative blood loss was 450 mL (300–750 mL). Blood volume was calculated at 80 mL/kg body weight. The mean blood loss as percentage of total volume was 18.0% (12.4–25.2%). The hemivertebrae types were 14 fully segmented, 10 semi-segmented, and three unsegmented (Table 1). None of the patients had preoperative neurologic dysfunction. Of the 27 patients, seven had open triangular cartilage, and 20 patients had closed triangular cartilage. The shortest fusion segment was three, the longest fusion segment was six, and the average fusion segment was 4.4.

**Table 1:** Demographics of the 27 study patients

Demographic	
Age (years)	11 (5–16)
Sex (male/female)	11 (40.7)/16 (59.3)
Follow-up (months)	68 (60–122)
Blood loss (mL)	450 (300–750)
Blood loss as percentage of total volume (%)	18.0% (12.4–25.2%)
Duration of surgery (minutes)	161 (60–300)
Type	
Fully segmented	14 (51.9)
Semi-segmented	10 (37.0)
Unsegmented	3 (11.1)
Level	
T11	11 (40.7)
T12	3 (11.1)
L1	4 (14.8)
L2	9 (33.3)

Values are mean (range) or number of patients (%)

*L* lumbar vertebrae, *T* thoracic vertebrae

### Radiographic deformity correction

The corrections in the coronal and sagittal planes are presented in Table 2.

**Table 2:** Coronal and sagittal plane correction

Radiographic	Preoperative	Postoperative	CR (%)	2 year follow-up	CR (%)	Final follow-up	CR (%)	*F*	*P*
Segmental scoliosis	40.35 ± 12.54 (19.3–69.6)^a^	11.94 ± 7.97 (1.0–28.8)^b^	71.2	12.77 ± 7.98 (2.1–36.0)^b^	67.4	13.24 ± 7.94 (3.8–40.0)^b^	65.9	122.692	< 0.001
Total main scoliosis	43.39 ± 14.46 (15.0–71.7)^a^	14.13 ± 9.33 (3.2–31.7)^b^	65.7	14.37 ± 8.66 (2.9–38.9)^b^	65.0	16.06 ± 10.19 (4.0–51.4)^b^	60.2	85.095	< 0.001
Compensatory caudal curve	15.78 ± 14.70 (0–67.7)^a^	3.57 ± 4.62 (0–14.5)^b^	78.9	4.72 ± 5.99 (0–25.8)^b^	62.0	4.38 ± 4.72 (0–14.2)^b^	69.2	16.895	< 0.001
Compensatory cranial curve	13.21 ± 13.41 (0–50.4)^a^	6.83 ± 10.68 (0–36.4)^b^	69.2	7.43 ± 11.86 (0–45.3)^b^	46.6	7.65 ± 11.86 (0–45.3)^ab^	30.3	7.972	0.003
Trunk shift	8.45 ± 18.32 (− 28.37 to 48.72)	7.65 ± 20.81 (− 30.97 to 56.40)	–	4.25 ± 10. 43 (− 29.11 to 23.22)	–	3.78 ± 11.55 (− 27.98 to 25.70)	–	0.802	0.437
Shoulder balance	3 ± 1 (− 20 to 20)	0 ± 11 (− 18 to 30)	–	1 ± 8 (− 18 to 20)	–	2 ± 7 (− 16 to 17)	–	0.622	0.554
Segmental kyphosis	34.30 ± 24.80 (2.0–83.7)^a^	15.88 ± 13.54 (0–41.7)^b^	62.0	15.57 ± 15.21 (0–53.4)^b^	58.6	15.12 ± 14.78 (0–50.7)^b^	61.9	33.440	< 0.001
Sagittal balance	− 16.98 ± 36.22 (− 75.90 to 59.88)	− 7.64 ± 28.17 (− 95.00 to 32.13)	–	– 10.13 ± 23.19 (− 50.40 to 33.50)	–	– 8.71 ± 24.11 (− 62.91 to 32.00)	–	1.212	0.305

*CR* correction rate. Values are expressed as mean ± SD (range)

Data were marked by letter-marking method. No statistically significant differences were noted between groups that marked the same letter, but statistically significant differences were noted between groups that marked different letters

### Main curve correction

A significant correction (*p* < 0.001) in segmental scoliosis was observed from preoperative [40.35° (19.3°–69.6°)] to the final postoperative follow-up [11.94° (1.0°–28.8°)], with a correction rate of 65.9%. The total main scoliosis curve was corrected from 43.39° (15.0°–71.7°) to 16.06° (4.0°–51.4°) at the final follow-up with a correction rate of 60.2% (*p* < 0.001).

### Compensatory curve correction

The compensatory caudal curve was corrected from 15.78° (0°–67.7°) preoperatively to 3.57° (0°–14.5°) postoperatively. The correction was maintained at the final follow-up 4.38° (0°–14.2°), with a correction rate of 69.2% (*p* < 0.001). The compensatory cranial curve was corrected from 13.21° (0°–50.4°) preoperatively to 6.83° (0°–36.4°) postoperatively. The value at the 2 year and final follow-up was 7.43° (0°–45.3°) and 7.65° (0°–45.3°), respectively, with a correction rate of 30.3%. The correction in the compensatory cranial curve was significant between preoperative and postoperative and 2-year follow-up (*p* < 0.001), but a statistically significant difference was not observed between the preoperative and final follow-up (*p* > 0.001).

### Sagittal plane correction

The SK corrected from 34.30° (2.0°–83.7°) to 15.88° (0°–41.7°) postoperatively, and the correction was maintained at the final follow-up, with a correction rate of 61.9% (*p* < 0.001).

### Coronal and sagittal balance

The change in trunk shift from 8.45 mm (− 28.37 to 48.72 mm) preoperative to 3.78 mm (− 27.98 to 25.70 mm) at the final follow-up was not significant (*p* > 0.001). The sagittal balance improved from − 16.98 mm (− 75.90 to 59.88 mm) preoperatively to − 8.71 mm (− 62.91 to 32.00 mm) at the final follow-up, although the change was not significant (*p* > 0.001). The change in shoulder balance from 3 mm (− 20 to 20 mm) preoperatively and 2 mm (− 16 to 17 mm) at the final follow-up was not significant (*p* > 0.001).

### Postoperative complications and additional surgery

The mean length-of-stay in hospital was 7 days postoperatively, and all patients did not require pediatric intensive care. The postoperative complications were shown in Table 3. No major intraoperative complications were noted. No wound infection, major vascular injuries, pedicle fractures, or neurologic complications occurred in any patient. Implant migration occurred in two cases at the 2-year follow-up and required additional implant removals. During follow-up, two patients encountered postoperative curve progression. At 2-year follow-up, five patients developed PJK and four patients showed asymptomatic. Furthermore, two patients showed both PJK and adding-on phenomena (Table 3). These patients were treated with braces and did not require reoperation.

**Table 3:** Postoperative complications

Patients	Value
Implant migration	2 (7.4)
Wound infection	0
Major vascular injury	0
Neurologic deficit	0
Curve progressions	2 (7.4)
Adding-on phenomenon	4 (14.8)
Proximal junctional kyphosis	5 (18.5)
Pseudarthrosis	0
Crankshaft phenomenon	0

Values are number of the 27 patients (%)

## Discussion

This study is the first retrospective evaluation of posterior thoracolumbar hemivertebrae resection and short-segment fusion for the management of congenital scoliosis. All patients achieved satisfactory outcomes. Significant corrections were documented in segmental scoliosis and kyphosis of 65.9% and 61.9% at final follow-up, respectively, and were maintained during the final follow-up. The overall complication rate was 40.7%, with only two patients requiring additional surgery secondary to implant migration.

For surgical treatment of patients with scoliosis, we usually consider fusion within six segments as short-segment fixation, and fixation with more than six segments as long-segment fixation. Usually, the short-segment fixation is necessary, but it increases the risk of postoperative scoliosis progression and adding-on phenomenon. The first case of hemivertebra resection was reported by Royle in 1928 [14]. More recently, in 2001, Shono et al. reported 12 hemivertebra resection using single posterior approach with a correction rate in segmental scoliosis of 63.3% [15]. For patients with thoracolumbar hemivertebrae, Bollini et al. reported a mean postoperative improvement of 69.3% [16]. Nakamura et al. reported five cases of hemivertebrae resection with an improvement of 55.1% for three thoracolumbar hemivertebrae [17]. Ruf et al. reported posterior only resection of 12 thoracolumbar hemivertebrae as early as 2003 [16]. In this study, the segmental scoliosis correction rate (65.9%) was similar to those of previous studies. Deformities were satisfactorily corrected, and no significant losses occurred during follow-up, suggesting that short-segment pedicle screw fixation provides sufficient force for correction.

Statistically significant differences between preoperative and postoperative measurements was noted in the compensatory cranial and caudal curve corrections, however, no significant difference was evident at the final follow-up. The corrections were 62.9% postoperatively and 30.3% at the final follow-up, showing some loss of correction. However, the compensatory caudal curve corrections of 78.9% were maintained at the final follow-up (69.2%). Compensatory cranial curve correction has been reported from 64.1 to 80% and from 66.9 to 79.0% for compensatory caudal curves [18–21]. The final correction rate for compensatory cranial curves in this study was lower than that reported in previous studies and can correlated with the levels of hemivertebrae. In the thoracic region, the deformity correction was resisted by the presence of deformed ribs and chest wall, whereas in the lumbar region, the vertebrae and ligaments were unable to resist the correction.

Hemivertebrae usually result in kyphotic deformity. The correction of the sagittal profile is as important as the correction in the coronal plane. Ruf et al. studied patients with congenital scoliosis who were treated with hemivertebra resection [18]. The SK improved from 22° to 8°. The correction rate of SK was 63.6%. In other studies, the correction rate of SK ranged from 63 to 81.9% [6, 8, 20, 22]. In this study, SK improved with a correction rate of 61.9% at the final follow-up, which was consistent with the findings of previous studies. These data suggest that the posterior hemivertebra resection and short-segment fusion reliably correct SK.

Coronal and sagittal balance are also major concerns in scoliosis. Corrections of trunk shift and sagittal balance have been reported variably in the literature. In this study, no significant differences between preoperative and final follow-up measurements were noted in the trunk shift and sagittal balance. Guo et al. [20] and Huang et al. [21] reported improvements in Coronal and sagittal balance, however, statistically significant difference was not observed. Li et al. [23] and Zhuang et al. [24] reported significant improvement in both coronal and sagittal balance. The reason for this difference is that the hemivertebrae in the studies by Li and Zhuang were located in the lumbosacral region [22, 23]. At the lumbosacral level, a minimal scoliosis curve can generate a substantial trunk shift. In the present study, the values of coronal and sagittal balance were less influenced by the thoracolumbar hemivertebrae and surgery. Posterior thoracolumbar hemivertebra resection and short-segment fusion gave limited improvement on coronal and sagittal balance.

Shoulder balance is an important index for evaluating coronal balance. To these authors’ knowledge, only one study evaluated shoulder balance after hemivertebra resection. Ruf et al. reported the shoulder imbalance of 9 mm preoperatively, 7 mm postoperatively, and 5 mm at final follow-up [10]. In this study, shoulder balance improved from 3.36 mm preoperatively to 1.65 mm at final follow-up; however, no significant differences were noted. The present study patients had better shoulder balance than in the Ruf study because their hemivertebrae were thoracolumbar, so the proximal hemivertebra compensation avoided the appearance of severe shoulder imbalance. These results showed limited improvement in shoulder balance following thoracolumbar hemivertebra resection.

Despite successful results of surgery, postoperative complications pose substantial concerns for this patient population. In this study, two patients (7.4%) experienced postoperative curve progression during follow-up. Bollini et al. [16] and Holte et al. [4] reported curve progression of 17.6% and 16.2% after hemivertebra resections, respectively. Short-segment fixation corrected the deformity and reduced the influence of adjacent vertebral growth. However, if the fusion segments are too short, curve progression may occur postoperatively. Incomplete hemivertebra resection is also associated with the main curve progression. Congenital anomalies have the potential for abnormal growth of vertebra and progress despite removal; these factors may in turn influence the structural differentiation into the adjacent vertebra. For these reasons, the remnant hemivertebra after operation may influence the main curve primarily and compensatory curves. The hemivertebra should be completely excised, and the appropriate fusion segments should be selected during surgery.

The main complication in this series was PJK, which often occurs in adults and adolescents following spinal fusion. In patients who are treated with posterior hemivertebra resection, PJK remains a major concern. Zhang et al. reported that one of 56 patients experienced PJK among those who underwent a one-stage posterior hemivertebra resection [25]. Wang et al. reported that 18.9% of patients (seven of 37) who underwent posterior hemivertebra resection and short-segment fusion experienced PJK [26]; Chen et al. reported a rate of 11.6% (22 of 189 patients) [27]. In the present study, the incidence of PJK was 18.5% (five of 27). This result was similar to those of previous studies.

Wang et al. analyzed the possible causes of PJK; the first and main point is that the misplaced screws in the upper instrumental vertebra may have disturbed the normal growth on the endplate [26]. The second point is that the excess damage caused in facet capsules or supraspinous ligaments above the fusion segments during exposure may be directly related to PJK. We also concluded that PJK was more common in patients with hemivertebra located in the lower thoracic or thoracolumbar regions. Taken together, these findings suggest that for patients with thoracolumbar hemivertebra, surgeons should pay more attention to the accuracy of screw placement and minimize damage to the surrounding soft tissues during surgery.

At 2-year follow-up, two patients were fixedly transferred within the patient’s appearance, and the imaging examination results showed that the patient’s hemivertebral resection segment had reached bony fusion, so internal fixation was taken out of the patient. The patient’s deformity did not progress further after the internal fixation was removed. Adding-on phenomenon is common in patients with adolescent idiopathic scoliosis. However, it can also occur after surgery for congenital scoliosis. Only two studies reported the adding-on phenomenon after hemivertebra resection. In 2015, Chang et al. first reported the adding-on phenomenon after hemivertebra resection, in which 11.1% of patients (two of 18) showed distal adding-on phenomenon [9]. Subsequently, in 2016, they reported 11.1% of patients (five of 45) showed distal adding-on phenomenon after hemivertebra resection [28]. In the present study, the incidence of adding-on was 14.8% (four of 27 patients), which was slightly higher than reported in these previous studies. The cause of the distal adding-on phenomenon is unclear. It may be a compensatory change for maintaining a well-balanced spine such as a balanced shoulder level, truncal shift, or listing. For patients with thoracolumbar hemivertebra after hemivertebra resection and short-segment fusion, the high mobility of the lumbar region makes this compensatory change more likely to occur.

The goal in the treatment of congenital scoliosis is to achieve a straight spine with a physiologic sagittal profile with as short a fusion segment as possible. Delayed treatment of an advanced deformity in older children or adults, however, must include the secondary structural curves and therefore requires long fusion segments. Furthermore, correction of these rigid curves is more difficult and associated with a higher risk of neurologic compromise. Progression of the curve is most rapid during the adolescent growth spurt, and progression stops only at skeletal maturity. Spontaneous neurologic deterioration due to compression of the spinal cord may occur in the case of congenital kyphoscoliosis, which generally manifests during the adolescent growth spurt at a mean age of 13.7 years [29]. Early and complete correction of the local deformity mitigates the development of secondary changes. Delayed treatment of an advanced deformity always requires an extensive and complex correction procedure. To date, however, there is no agreement regarding the appropriate surgical window, despite the fact that most clinicians suggest surgery as early as possible after 1 year of age. However, patients with congenital scoliosis who visited our spine center for the first time are often over 7 years old. For these patients, after the diagnosis of scoliosis, we performed the surgical treatment [30]. Although they are older, they were satisfied with the clinical efficacy. In this study, eight patients were younger (age < 7 years) and 19 patients were older (age > 7 years).

This study has several limitations. Follow-up did not include quality of life. Further trials are needed to analyze a correlation between radiological correction and clinical outcome (Scoliosis Research Society scores-24, Oswestry Disability Index). All radiologic measurements were performed by one independent radiologist; although this design may introduce single observation error, the results were consistent with values in the literature. This study reviewed only postoperative complications, and the small number of study patients may overestimate the rate of complications. Further studies are needed to analyze the causes and risk factors of these complications.

## Conclusion

Posterior thoracolumbar hemivertebra resection and short-segment fusion effectively treat congenital scoliosis. The efficacy of correction is demonstrated by radiographic measurements. Long-term follow-up revealed good maintenance of correction. However, postoperative complications such as curve progression, PJK, and the adding-on phenomenon may cause substantial concern among patients and surgeons.

## Data Availability

The datasets generated and/or analyzed during the current study are not publicly available due to the data being confidential; however, they are available from the corresponding author on reasonable request.

## Abbreviations

SK: Segmental kyphosis
RSH: Radiographic shoulder height
CSVL: Center sacral vertical line
PJK: Proximal junctional kyphosis

## References

[1] Kotwicki T, Negrini S, Grivas TB, Rigo M, Maruyama T, Durmala J, Zaina F (2009). Methodology of evaluation of morphology of the spine and the trunk in idiopathic scoliosis and other spinal deformities—6th SOSORT consensus paper. Scoliosis.

[2] Winter RB, Moe JH, Lonstein JE (1984). Posterior spinal arthrodesis for congenital scoliosis. An analysis of the cases of two hundred and ninety patients, five to nineteen years old. J Bone Joint Surg Am.

[3] Akbarnia BA, Hosseini P. Expert’s comment concerning Grand Rounds case entitled "Selective hemivertebrae resection in a congenital scoliosis patient with multiple hemivertebrae deformities" by Yangpu Zhang et al. (Eur Spine J; 2017. 10.1007/s00586-017-4960-7). Eur Spine J. 2017;26(6):1584–1585. 10.1007/s00586-017-4999-5 28281002

[4] Holte DC, Winter RB, Lonstein JE, Denis F (1995). Excision of hemivertebrae and wedge resection in the treatment of congenital scoliosis. J Bone Joint Surg Am.

[5] Wang S, Zhang J, Qiu G, Wang Y, Li S, Zhao Y, Shen J, Weng X (2012). Dual growing rods technique for congenital scoliosis: more than 2 years outcomes: preliminary results of a single center. Spine.

[6] Dayer R, Ceroni D, Lascombes P (2014). Treatment of congenital thoracic scoliosis with associated rib fusions using VEPTR expansion thoracostomy: a surgical technique. Eur Spine J.

[7] McCarthy RE, McCullough FL (2015). Shilla growth guidance for early-onset scoliosis: results after a minimum of five years of follow-up. J Bone Joint Surg Am.

[8] Huang Y, Feng G, Liu L, Yang X, Song Y, Zhou C, Wang L, Zhou Z (2018). Posterior hemivertebral resection for upper thoracic congenital scoliosis: be aware of high risk of complications. J Pediatr Orthop B.

[9] Chang DG, Kim JH, Ha KY, Lee JS, Jang JS, Suk SI (2015). Posterior hemivertebra resection and short segment fusion with pedicle screw fixation for congenital scoliosis in children younger than 10 years: greater than 7-year follow-up. Spine.

[10] Ruf M, Harms J (2002). Hemivertebra resection by a posterior approach: innovative operative technique and first results. Spine.

[11] Wang Y, Hansen ES, Høy K, Wu C, Bünger CE (2011). Distal adding-on phenomenon in Lenke 1A scoliosis: risk factor identification and treatment strategy comparison. Spine.

[12] Lakhal W, Loret JE, de Bodman C, Fournier J, Bergerault F, de Courtivron B, Bonnard C (2014). The progression of lumbar curves in adolescent Lenke 1 scoliosis and the distal adding-on phenomenon. Orthop Traumatol Surg Res.

[13] Mika AP, Mesfin A, Rubery PT, Molinari R, Kebaish KM, Menga EN (2019). Proximal junctional kyphosis: a pediatric and adult spinal deformity surgery dilemma. JBJS Rev.

[14] Royle ND (1928). The operative removal of an accessory vertebra. Med J Aust.

[15] Shono Y, Abumi K, Kaneda K (2001). One-stage posterior hemivertebra resection and correction using segmental posterior instrumentation. Spine.

[16] Bollini G, Docquier PL, Viehweger E, Launay F, Jouve JL (2006). Thoracolumbar hemivertebrae resection by double approach in a single procedure: long-term follow-up. Spine.

[17] Nakamura H, Matsuda H, Konishi S, Yamano Y (2002). Single-stage excision of hemivertebrae via the posterior approach alone for congenital spine deformity: follow-up period longer than ten years. Spine.

[18] Ruf M, Jensen R, Letko L, Harms J (2009). Hemivertebra resection and osteotomies in congenital spine deformity. Spine.

[19] Wang S, Zhang J, Qiu G, Li S, Yu B, Weng X (2013). Posterior hemivertebra resection with bisegmental fusion for congenital scoliosis: more than 3 year outcomes and analysis of unanticipated surgeries. Eur Spine J.

[20] Guo J, Zhang J, Wang S, Zhang Y, Yang Y, Yang X, Zhao L (2016). Surgical outcomes and complications of posterior hemivertebra resection in children younger than 5 years old. J Orthop Surg Res.

[21] Huang Y, Feng G, Song Y, Liu L, Zhou C, Wang L, Zhou Z, Yang X (2017). Efficacy and safety of one-stage posterior hemivertebral resection for unbalanced multiple hemivertebrae: a more than 2-year follow-up. Clin Neurol Neurosurg.

[22] Ruf M, Harms J (2003). Posterior hemivertebra resection with transpedicular instrumentation: early correction in children aged 1 to 6 years. Spine.

[23] Li Y, Wang G, Jiang Z, Cui X, Li T, Liu X, Zhang W, Sun J (2017). One-stage posterior excision of lumbosacral hemivertebrae: retrospective study of case series and literature review. Medicine (Baltimore).

[24] Zhuang Q, Zhang J, Li S, Wang S, Guo J, Qiu G (2015). One-stage posterior-only lumbosacral hemivertebra resection with short segmental fusion: a more than 2-year follow-up. Eur Spine J.

[25] Zhang J, Shengru W, Qiu G, Yu B, Yipeng W, Luk KD (2011). The efficacy and complications of posterior hemivertebra resection. Eur Spine J.

[26] Wang Y, Kawakami N, Tsuji T, Ohara T, Suzuki Y, Saito T, Nohara A, Tauchi R, Kawakami K (2017). Proximal junctional kyphosis following posterior hemivertebra resection and short fusion in children younger than 10 years. Clin Spine Surg.

[27] Chen X, Xu L, Qiu Y, Chen ZH, Zhu ZZ, Li S, Sun X (2018). Incidence, risk factors, and evolution of proximal junctional kyphosis after posterior hemivertebra resection and short fusion in young children with congenital scoliosis. Spine.

[28] Chang DG, Yang JH, Lee JH, Kim JH, Suh SW, Ha KY, Suk SI (2016). Congenital scoliosis treated with posterior vertebral column resection in patients younger than 18 years: longer than 10-year follow-up. J Neurosurg Spine.

[29] McMaster MJ, Singh H (1999). Natural history of congenital kyphosis and kyphoscoliosis. A study of one hundred and twelve patients. JBJS.

[30] Klemme WR, Polly DW, Orchowski JR (2001). Hemivertebral excision for congenital scoliosis in very young children. J Pediatr Orthop.

